# Immunosuppressive Therapy for Usual Interstitial Pneumonia in Autoimmune Rheumatic Diseases: A Review

**DOI:** 10.3390/medicina61040599

**Published:** 2025-03-26

**Authors:** Domenico Sambataro, Giulia Morina, Alessandro Libra, Stefano Palmucci, Francesco Pallotti, Giulio Geraci, Gaetano La Rocca, Francesco Ferro, Michele Moretti, Chiara Baldini, Carlo Vancheri, Gianluca Sambataro

**Affiliations:** 1 Outpatient Clinic Associated with the Regional Health System, Artroreuma s.r.l., 95030 Mascalucia (CT), Italy; d.sambataro@hotmail.it; 2 Department of Clinical and Experimental Medicine, Regional Referral Center for Rare Lung Diseases, Policlinico “G.Rodolico-San Marco”, University of Catania, 95123 Catania, Italy; giulia.morina@tiscali.it (G.M.); alessandrolibra@outlook.it (A.L.); vancheri@unict.it (C.V.); 3 Department of Medical Surgical Sciences and Advanced Technologies “GF Ingrassia”, University Hospital Policlinico “G.Rodolico-San Marco”, Unità Operativa Semplice Dipartimentale di Imaging Polmonare e Tecniche Radiologiche Avanzate (UOSD IPTRA), University of Catania, 95123 Catania, Italy; spalmucci@unict.it; 4 Department of Medicine and Surgery, University of Enna “Kore”, 94100 Enna, Italy; francesco.pallotti@unikore.it (F.P.); giulio.geraci@unikore.it (G.G.); 5 Rheumatology Unit, Department of Clinical and Experimental Medicine, University of Pisa, 56126 Pisa, Italy; gaelarocca94@gmail.com (G.L.R.); francescoferrodoc@gmail.com (F.F.); michele.moretti93.md@gmail.com (M.M.); chiara.baldini74@gmail.com (C.B.)

**Keywords:** idiopathic pulmonary fibrosis, rheumatoid arthritis, treatment, systemic sclerosis, usual interstitial pneumonia, fibrosis, interstitial lung disease, anti-synthetase syndrome, idiopathic inflammatory myopathies, Sjögren’s syndrome

## Abstract

Usual Interstitial Pneumonia (UIP) is the most severe radiological/histological pattern of Interstitial Lung Disease (ILD). It is typical of Idiopathic Pulmonary Fibrosis (IPF), but is also frequently described in Autoimmune Rheumatic Diseases (ARDs), sharing with IPF common risk factors, genetic backgrounds, and in some cases, disease progression and prognosis. Following the results of the PANTHER study, immunosuppressive drugs are now not recommended for the treatment of IPF; however, their use for the treatment of UIP secondary to ARDs is still under debate. The aim of this review is to summarize existing knowledge on the clinical presentation of autoimmune UIP and its treatment with immunosuppressive drugs. We searched PubMed for English language clinical trials and studies on treatment of ARDs-ILD, looking for specific treatments of UIP-ARDs. The available clinical trials rarely stratify patients by ILD pattern, and clinical studies generally lack a comparison with a placebo group. In Systemic Sclerosis, UIP patients showed a non-significant trend of worsening under immunosuppression. On the contrary, in Interstitial Pneumonia with Autoimmune Features and, above all, Rheumatoid Arthritis, immunosuppressive treatment produced promising results in the management of UIP patients. In conclusion, the current evidence about the immunosuppressive treatment of UIP-ARDs is limited and conflicting. There is an urgent need to adequately assess this topic with specific clinical trials, as has already been performed for IPF. The possibility should be considered that different ARDs can respond differently to immunosuppression. Finally, a wider use of histological samples could produce valuable information from a diagnostic, therapeutic, and research point of view.

## 1. Introduction

The term “Interstitial Lung Disease” (ILD) refers to a condition characterized by the deposition of inflammatory cells and/or extracellular matrix in the lung interstitium, potentially leading to progressive respiratory failure and, ultimately, death [1]. ILD can present with consolidations, Ground Glass Opacities (GGOs), reticulation, or fibrosis, and, according to the distribution of these items, has been classified into three main HRCT patterns: Organizing Pneumonia (OP), Nonspecific Interstitial Pneumonia (NSIP), and Usual Interstitial Pneumonia (UIP), whereas in the absence of specific signs, the radiological pattern can be considered indeterminate [2]. Over 200 clinical entities are associated with ILD, the most common being Idiopathic Pulmonary Fibrosis (IPF), Connective Tissue Diseases (CTDs)–ILD, and Hypersensitivity Pneumonia, which account for 30%, 25%, and 15% of total ILDs, respectively [1]. IPF, characterized by a progressive fibrosis of the lungs, in the context of a UIP radiological and histological pattern, is the most common condition and is also associated with the poorest prognosis [3].

The UIP pattern presents histologically with areas with confluent and dense scarring of the alveolar parenchyma mixed with spared, normal areas, interrupted by the foci of fibroblasts [4]. From a radiological point of view, a UIP pattern is characterized by a subpleural or basal predominance of Honeycombing (HC), defined by the presence of clustered cystic airspaces of 3–10 mm with thick walls, and accompanied by reticulation and possible traction bronchiectasis or bronchiolectasis [2]. Some grades of GGO may be present but are not significant. Ossified nodules can be present in about 30% of patients [2]. Histological and radiological features show a high concordance with each other, reaching 90–100% [5]. A similar radiological pattern where HC is not detected is defined as “probable UIP”, and, in the appropriate clinical setting, often reflects a histological UIP pattern, considering that histology precedes the radiological features [6].

The UIP pattern is not pathognomonic of IPF: it is the most common pattern of ILD associated with Rheumatoid Arthritis (RA) and vasculitides, but is also frequently reported in other Connective Tissue Diseases (CTDs) such as longstanding Systemic Sclerosis (SSc) and primary Sjögren’s syndrome (pSS), in which it can precede the onset of typical sicca syndrome [7,8,9]. Finally, it is also described in anti-synthetase syndrome (ASyS), mainly in non-Anti-Jo1-antibody-positive subsets [10].

The radiological UIP pattern associated with autoimmune diseases can be distinguished from IPF by the presence of HC in the anterior part of the upper lobe (upper lobe sign), of exuberant HC (more than 70% of the fibrotic lung areas), or by the presence of the straight-edge sign (basal fibrosis with horizontal demarcation from the spared upper and midzones) [11]. Histologically, a UIP pattern associated with ARDs can show unexplained pleural inflammation, nodular aggregates of lymphocytes with or without germinal centers, lymphocyte infiltration in the normal parenchyma, or organizing pneumonia determining mixed patterns [11].

Despite these clues, IPF-associated and ARDs-associated UIP patterns cannot be easily distinguished, suggesting that different multi-step pathogenic mechanisms tend to converge towards a common pathway driven by myofibroblasts. The identification of the condition underlying UIP-ILD currently drives treatment, given that the idiopathic form is treated with anti-fibrotics, whereas the common approach to all ARDs-ILD is based on their pathogenesis, immunosuppression, plus anti-fibrotics in the case of proven progressive phenotypes.

However, a crucial question is whether treatment should be driven by histologically and/or radiologically proven lung damage, or by the underlying pathogenesis.

This review aims to summarize the current evidence on immunosuppressive therapy for UIP-ILD, with a focus on those associated with ARDs, to identify current research gaps. Its design as a scoping review is, in our opinion, justified by the different conditions evaluated and the heterogeneity of the studies reviewed.

## 2. Methods

We performed a literature search in January–February 2025 on PubMed, looking for clinical studies and clinical trials in the English language about the treatment of ARDs associated with a UIP pattern. We used the MeSH terms “rheumatoid arthritis”, “scleroderma”, “systemic sclerosis”, “idiopathic inflammatory myopathies”, “polymyositis”, “dermatomyositis”, “Sjögren’s syndrome”, “anti-synthetase syndrome”, “mixed connective tissue disease”, “connective tissue disease”, “autoimmune disease”, “vasculitis”, “granulomatosis with polyangiitis”, “micro polyangiitis” AND “interstitial lung disease”, “lung fibrosis”, “pulmonary fibrosis”, AND “treatment”, “therapy”, “abatacept”, “mycophenolate”, “methotrexate”, “azathioprine”, “leflunomide”, “cyclosporine A”, “tacrolimus”, “cyclophosphamide”, “rituximab”, “tocilizumab”, “filgotinib”, “baricitinib”, “Upadacitinib”, “tofacitinib”. The selected articles were evaluated using our eligibility criteria and reviewed for possible citations of similar articles not identified by our selection.

The eligibility criteria were presence on PubMed, written in the English language, and being clinical trials OR clinical studies in which at least one sub-analysis of UIP patients was conducted with the use of immunosuppressive drugs. Case reports and case series on less than five patients were excluded from our analysis. D.S., G.S., and C.V. conducted the article selection process. The main variables considered were mortality and changes in Forced Vital Capacity (FVC).

The manuscripts selected were read carefully, with the additional aim of identifying similar articles not found by the MeSH terms used. The study protocol was not registered.

The literature search produced 528 different results, of which 22 were excluded due to their nature (case report/series or review), 342 because the articles were outside the manuscript’s scope, 115 because the UIP numbers were not reported or were insufficient, and 35 for other reasons (treatment or ILD pattern unclear, different languages used). A total of 14 manuscripts were taken into consideration.

## 3. Pathogenesis of Lung Fibrosis

Most of the studies analyzing the pathogenesis of lung fibrosis were focused on IPF. Repeated lung epithelial damage to Type I Alveolar Epithelial Cells (AEC1) and repair dysfunction in Type II Alveolar Epithelial Cells (AEC2) are the primers of the disease [12]. Environmental exposure to dust and smoking habits are known risk factors for the development of lung damage, but further data also support the role of infections and local microbiota [12,13]. Moreover, predisposing genetic and epigenetic factors are also involved, such as mutations to genes responsible for the maintenance of telomere length and epithelial barrier function (e.g., TERT), the inhibition of Transforming Growth Factor β (TOLLIP), and, above all, MUC5B mutation. Aging is associated with a reduction in telomere length, leading to defects in stem cell renewal, abnormal DNA repair and, potentially, the direct promotion of fibrosis [14]. Other epigenetic alterations to DNA methylation lead to the production of different miRNA, eventually causing the impairment of apoptosis and an increase in fibrotic processes.

**Figure 1:**
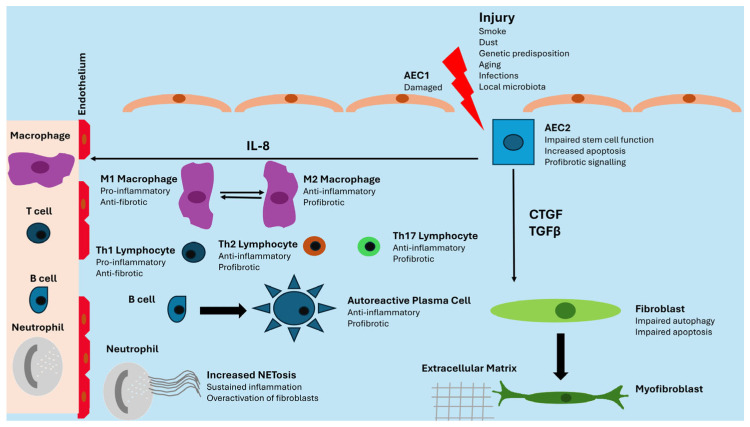
Schematic of pathogenesis of lung fibrosis. AEC: Alveolar Epithelial Cell; CTGF: Connective Tissue Growth Factor; IL-8: Interleukin 8; TGFβ: Transforming Growth Factor β.

AEC2 cells are pressed into service to renew injured AEC1 cells, but the genetic and epigenetic alterations previously described lead to impaired stem cell function, apoptosis, and pro-fibrotic signaling mediated by Transforming Growth Factor β (TGFβ), Connective Tissue Growth Factor (CTGF), and IL8. TGFβ and CTGF favor the transition of fibrocytes and fibroblasts into myofibroblasts, while IL8 has a chemoattractant action on innate immunity cells. Lung fibroblasts show impaired autophagy and apoptosis, two mechanisms involved in the control of damaged and senescent cells. Under continuous profibrotic signals, they expand prolifically and differentiate into myofibroblasts, with an increased capacity for producing extracellular matrix [15,16]. The immune system plays a controversial role. During the first stages, M1-polarized macrophages show anti-fibrotic properties, but in the second phase, M2-polarized macrophages display fibrotic activity. Neutrophils are recruited in the damaged lung and favor fibrosis through NETosis, whereas adaptive immunity is characterized by the production of pro-inflammatory and anti-fibrotic cytokines by Th1 lymphocytes in the first stages and by the secretion of anti-inflammatory and pro-fibrotic mediators by Th2 and Th17 lymphocytes in the established disease. Active B lymphocytes also produce autoantibodies against lung self-antigens [17]. The immune system may play different roles in lung fibrosis when it is associated with idiopathic or secondary forms [11]. Figure 1 summarizes these processes schematically.

## 4. Immunosuppressive Therapy in Idiopathic Pulmonary Fibrosis

During the early 2000s, the major rationale driving the treatment of IPF was the progression of lung inflammation towards fibrosis. The guidelines, although very cautiously, suggested at least one attempt at therapy with prednisone and/or Azathioprine [18]. Prednisone was indicated as being a potential initial treatment, reserving Azathioprine for refractory disease or as a steroid-sparing agent. Prednisone therapy, despite being used empirically in many centers, has never been evaluated in clinical trials. A small but transient improvement was noted in 10–30% of patients [18,19]. Use of Azathioprine, alone or accompanying a low dose of steroids, was supported by a small clinical trial reporting a trend towards increased survival in patients treated with Azathioprine plus prednisone compared to prednisone plus placebo [20]. This limited evidence led to a larger scale evaluation of a combined treatment called “triple therapy”, which included Azathioprine, Prednisolone, and N-acetylcysteine. In the study, conducted by Demendts M. et al., “triple therapy” was shown to significantly slow down deterioration of pulmonary function compared with Azathioprine plus Prednisolone alone, with similar adverse effects but also with a similar mortality (9% and 11%, respectively) [21]. The major limitation of this clinical trial was the absence of a real placebo group, as proven by another clinical trial, named “the PANTHER study”, which was specifically conducted to evaluate the safety and effectiveness of triple therapy versus N-acetylcysteine alone and placebo alone [22]. The triple therapy arm was terminated because of an increased mortality rate in the triple therapy group compared with placebo (11% vs. 1%), mainly due to respiratory infections, whereas the N-acetylcysteine-alone arm did not show any difference compared with placebo [23].

It should be noted that patients enrolled in the trial by Demendts M. et al. [21] were similar in terms of their general features, disease duration, and pulmonary function to those enrolled in the PANTHER study. Even the mortality rate in the treated groups was similar (11%), allowing us to presume that the presence of an actual placebo group in the first trial would also have had similar results.

Despite the disappointing results of these clinical trials, empirical experience and previous studies have suggested a potential role for immunosuppressive treatment in IPF, influencing the approach to IPF therapy in daily clinical practice.

## 5. Diagnosis Challenges: The Example of Rheumatoid Arthritis

UIP is the most common pattern of ILD in RA, affecting about 55% of RA-ILD patients [7]. Among the risk factors associated with the development of ILD in RA, some are also shared with IPF, such as male gender, older age, a history of smoking, and MUC5B variants, whereas others are disease-specific, such as Rheumatoid Factor and Anti-Citrullinated Protein Antibody (ACPA) positivity, older age at diagnosis, and higher disease activity [24,25]. However, most of the studies on RA-ILD were performed in rheumatological settings. Patients with severe lung involvement may initially be referred to respiratory units, and in this case, diagnosis is often challenging. A recent study compared the clinical presentation of RA patients diagnosed in a rheumatological setting to those diagnosed in an ILD unit. Those patients had similar seropositivity rates and levels of acute phase reactants, but patients diagnosed in a respiratory setting showed a trend towards increased erosions and a presentation resembling Polymyalgia Rheumatica (PMR) in about 48% of cases, compared to 13% of cases in rheumatological settings [26]. These data are consistent with the expected clinical presentation of elderly onset RA, characterized by increased bone erosions and higher mortality rates [27].

**Figure 2:**
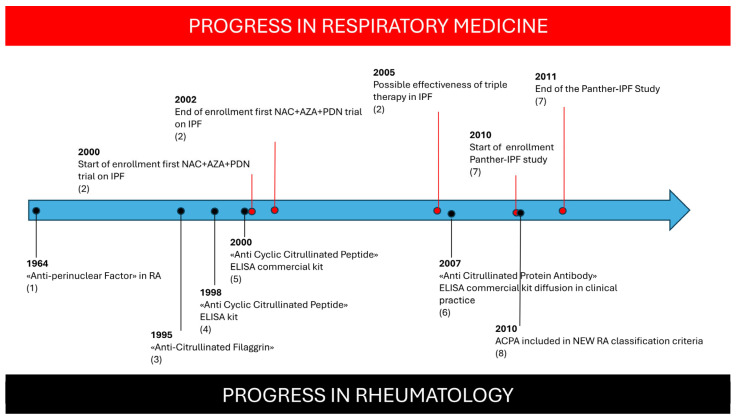
Progression of respiratory trials compared with progress in knowledge about ACPA autoantibodies in Rheumatoid Arthritis. Figure Caption: (1): Nienhuis RL et al., Ann Rheum Dis 1964 [29]; (2): Demendts N et al., New Eng J Med 2005 [21]; (3): Sebbag N et al., J Clin Invest 1995 [30]; (4): Schellenkens GA et al., J Clin Invest 1998 [31]; (5): Schellenkens GA et al., Arthritis Rheum 2000 [32]; (6): Coenen D et al., Clin Chem 2007 [33]; (7): PANTHER Study, New Eng J Med 2012 [22]; (8): Aletaha D et al., Ann Rheum Dis 2010 [34].

Polymyalgia Rheumatica is characterized by severe morning stiffness of the shoulder and hip girdles which is associated with severe inflammation without peripheral joint swelling. In seronegative patients presenting with PMR, the misclassification of RA-ILD as IPF is understandable, given their similar risk factors, radiological pattern, and even progression of lung damage [28]. The identification of ACPA is crucial for RA diagnosis: the progressive spread of ACPA testing in routine clinical practice since the inclusion of ACPA in the 2010 classification criteria for RA has greatly increased sensitivity in RA diagnoses in different settings [29,30,31,32,33,34]. The improved recognition of RA features in respiratory settings may have influenced the enrollment of patients in the PANTHER study, leading to a more homogeneous cohort of IPF patients and a more reliable result (Figure 2). Conversely, the small proportion of UIP patients with an increased survival rate reported in previous studies [18,19] could be accounted for by an underlying autoimmune origin.

## 6. UIP in Systemic Autoimmune Rheumatic Disease

UIP is the most frequent ILD pattern associated with Anti-Neutrophil Cytoplasmic Antibody (ANCA)-associated vasculitides. This group includes Microscopic Polyangiitis (MPA), Eosinophilic Granulomatosis with Polyangiitis (EGPA), both associated with p-ANCA and anti-MPO positivity, and Granulomatosis with Polyangiitis (GPA), which is associated with the presence of c-ANCA and anti-PR3. ILD is present in up to 45% of cases of MPA and 23% of GPA. The radiological and histological UIP pattern is reported in 48–100% cases of ILD associated with ANCA–vasculitides. As already highlighted for RA and IPF, the UIP-ILD is mainly described in male patients over 65 years of age, mostly associated with a smoking habit. Also, in ANCA-ILD, diagnosis can be challenging, because ILD can precede the typical manifestations of vasculitides by years in 14–85% of patients; the prevalence of ANCA in patients initially presented with IPF ranges between 4 and 36% for MPO-ANCA and 2–4% for PR3-ANCA; and even in histological lung samples, despite the possible presence of NSIP features as a minor associated pattern, signs of vasculitis and capillaritis are rare [35].

Most of the studies on CTD-associated ILD are performed on SSc subjects. The typical radiological ILD pattern in this condition is a fibrotic NSIP. Biopsies are not routinely performed on SSc for clinical or research purposes. The limited studies demonstrate a poor correlation between HRCT and histologic findings, given that the NSIP radiological pattern changed to a histological UIP in up to a third of SSc patients, mainly in the limited cutaneous subset of the disease [36]. The radiological UIP-like pattern is also common in SSc diagnosed in respiratory settings, associated with an unusual seronegativity for specific SSc-related autoantibodies in 20% of cases [26].

In Idiopathic Inflammatory Myopathies (IIMs), the most common radiological pattern of ILD is cellular NSIP with or without features of OP [37]. However, studies reporting histologic data are limited, and commonly enroll small cohorts of patients. Dermatomyositis patients showed a histologic UIP pattern in 5–20% of cases [38,39,40,41], whereas ASyS showed a histologic UIP pattern in about 25% of cases, with a mild prevalence of non-Jo1+ cases [42]. Diagnosing underlying IIM in UIP patients in respiratory settings can be difficult, considering that patients who are seropositive for autoantibodies different from Jo1 show a clinical picture in which ILD is the first, and often only, clinical manifestation [43]. Given that the other anti-synthetase autoantibodies are not tested in the most common commercial kits for Extractable Nuclear Antigens, those autoantibodies can remain unrecognized until further clinical features suggesting an underlying IIM appear.

Similarly to IIM-ILD, pSS-ILD is commonly associated with an NSIP ± OP pattern, despite the potential presence of a Lymphocytic Interstitial Pneumonia pattern, which is very rare but highly specific for pSS [7]. The UIP pattern is described in about 20% of cases both radiologically and histologically [7,44]. ILD can precede the diagnosis of pSS by up to ten years. In this case, ILD shows a UIP radiological pattern in 56% of patients, associated with no or very mild sicca symptoms and commonly associated with seronegativity. These patients are clearly always diagnosed with IPF until specific autoimmunity features are demonstrated [45].

Finally, Undifferentiated CTD (UCTD) can also show a UIP pattern. The definition of UCTD in ILD patients is generally based on the Interstitial Pneumonia with Autoimmune Features (IPAF) criteria proposed in 2015 [46]. The presence of at least one clinical or serological feature in the presence of an NSIP, OP, or LIP pattern, or otherwise, in the case of UIP, at least one clinical and one serological feature, are required to classify a patient as having IPAF. This distinction is made to limit the classification of IPAF in patients with a UIP pattern, which is deemed to not be strongly associated with CTD-ILD. Therefore, it is not surprising that prospective studies on IPAF reported the prevalence of a UIP pattern that was lower than 10% [47]. However, other studies showed that, when associating only one domain with the UIP pattern, those patients diagnosed with IPAF had a similar rate of progression towards specific autoimmune diseases compared to “classic” UIPAF, greater than the rate reported in IPF [48,49]. For these reasons, the current distinction regarding UIP patients is under debate.

In conclusion, the UIP pattern is associated with an autoimmune disease in a non-negligible proportion of patients, and recognition of the underlying ARD is challenging because of the different clinical presentations and relatively common seronegativity. However, whether this subset of UIP patients could benefit or not from immunosuppressive treatments is the crucial topic.

## 7. Immunosuppressive Treatment of Secondary UIP

Treating UIP patients with immunosuppressants is a matter of debate. Currently, trials are conducted mainly on SSc patients. In Scleroderma Lung Study I, intravenous Cyclophosphamide was superior to Azathioprine (AZA), but its advantage was not maintained at a 2-year-follow up [50,51], whereas in Scleroderma Lung Study II, oral Cyclophosphamide and Mycophenolate Mofetil (MMF) exhibited a similar efficacy to Cyclophosphamide, but with greater safety [52]. These results were not confirmed in terms of statistical significance in other clinical trials: a trial published the same year as Scleroderma Lung Study I evaluated the efficacy of low-dose Prednisolone and monthly infusions of Cyclophosphamide followed by AZA versus placebo, but failed to reach its primary and secondary outcomes, showing only a trend towards an improvement in FVC in the treated arm [53]. The trial regarding tocilizumab use evaluated only FVC as a secondary outcome, which resulted in stability on the treated arm [54]. Finally, a recent clinical trial analyzed Rituximab (RTX) versus intravenous Cyclophosphamide use in a cohort of CTD-ILD, mainly composed of IIM and SSc patients. The two drugs showed similar outcomes, but RTX was associated with a better safety profile [55]. None of these trials reported data about the enrollment of UIP patients. Another clinical trial on SSc ILD was Scleroderma Lung Study III, in which MMF + pirfenidone showed similar improvements in FVC to MMF + placebo (results available only as an abstract) [56]. In RA-ILD, a clinical trial aimed at evaluating abatacept is not currently available, while the PULMORA study, designed to evaluate tofacitinib vs. methotrexate, was terminated due to low enrollment [57,58].

Of particular interest for this manuscript is the NCT02896205 trial, in which 41 SSc-ILD patients were treated with MMF 2 g/day or placebo. No significant differences were found in respiratory outcomes, but the authors reported a sub-analysis of fifteen UIP patients (six treated with MMF and nine in the placebo arm): the median FVC change was −2.5% in the treated group and +3.5% in the placebo arm [59].

Beyond clinical trials, a retrospective study on fibrotic CTD-ILD, including 30% UIP patients, prompted by the results of the PANTHER study, tested the safety of treatment with AZA, showing no increase in mortality; however, the control group, which had similar features, was composed of patients treated with MMF rather than placebo [60]. Therefore, these data could have been affected by bias, much like the results of the IPF studies before the PANTHER study. Another retrospective study on the treatment of 32 histological or radiological UIP-CTD patients with MMF found a strong trend of a decline before treatment and a stabilization after MMF [61]. Similar results were found using RTX for the treatment of two small groups of CTD-ILD, in which the stabilization of FVC was demonstrated irrespective of the presence of a UIP pattern [62,63].

In SSc-ILD, a study used the same treatment with steroids and oral Cyclophosphamide for NSIP and UIP histological patterns, proving a trend of increased mortality for patients with a UIP pattern [36]. However, this could be explained by the intrinsically worse prognosis associated with a UIP pattern; also, in this case, the limited number of patients enrolled and the absence of a real placebo group meant that definitive conclusions could not be drawn.

In RA-ILD, the use of a biological treatment different to anti-TNF (abatacept, tocilizumab, RTX) was associated with the slower worsening of lung function irrespective of the presence of a UIP pattern [64]. Other studies on small groups of UIP-RA patients treated with RTX showed conflicting results: while in the study by Narvaez J et al., treatment significantly improved FVC in UIP patients [65], in other studies, UIP was considered a risk factor for increased mortality [66,67]. The study by Fui A. et al. found a decline of FVC of 301 mL in UIP patients treated with RTX vs. 51 mL in non-UIP patients who received treatments [67]. Also, in this case, no definitive conclusions could be drawn. Regarding new therapies, a recently published study on 78 UIP-RA patients treated with tofacitinib and iguratimod found a significant improvement in FVC and HRCT fibrosis scores compared with treatment with conventional DMARDs, while also improving joint disease activity [68]. These results were confirmed in another group of RA-ILD patients, including a UIP pattern treated with JAK inhibitors [69]. These data are promising, with a view to the possible treatment of this subgroup of patients, looking for a dual control of both lung and joint involvement. Finally, a recent study involving a bigger group of RA-UIP patients described a different trajectory of FVC worsening in patients treated with immunosuppressants, suggesting that some benefit may be derived from these treatments [70].

Similar results were obtained in IPAF, a condition in which the autoimmune profile can vary considerably between patients: IPAF patients with a UIP radiological pattern treated as IPF shared a similar prognosis, whereas another study on histological UIP-IPAF showed an improvement in FVC after immunosuppression and better survival in those patients treated with immunosuppressants compared with those treated with anti-fibrotics. The improvement was associated with the histological presence of inflammatory cell infiltration in the context of a UIP pattern [71,72]. 

Table 1 summarizes the studies reported.

## 8. Future Perspectives

Treating SARDs-associated UIP with immunosuppressive treatments is a topic which is almost unexplored, as no clinical trial has been specifically designed for this purpose. A subgroup analysis of the already cited trials has provided inconclusive results. Even the results in clinical trials regarding the use of nintedanib in SSc and progressive fibrosing ILD do not appear to be influenced by the presence of combined therapy with MMF [73,74].

Considering real-life experiences, conflicting results were reported: despite studies such as Manson et al. [70] suggesting a potential improvement in RA-UIP, other studies (mainly in SSc) reported a mortality comparable to that found in the PANTHER study. These data are interesting, considering that UIP-CTD is generally associated with a better prognosis compared to IPF [75]. Thus, the real question is whether immunosuppressive therapy in this specific group of patients could have any benefit or even be harmful, as has already been demonstrated in IPF. This is of particular interest, considering that those autoimmune diseases, even when characterized by UIP-ILD, may require immunosuppressive treatment for the management of other systemic manifestations, with a potential impact on lung disease, as described in RA, where the use of anti-TNFα blockers was associated with a potential increase in mortality linked to ILD [76].

As already suggested in previous studies [77], it is possible that different responses to immunosuppressive treatments in UIP patients may depend on different cellular infiltrates in the context of a histological UIP. This hypothesis should be supported by further larger and prospectively designed studies, in order to increase the number of lung samples obtained from CTD-ILD. At present, lung biopsy is performed for diagnostic purposes in doubtful clinical and radiological conditions. Patients with ARDs-associated ILD, at least in a rheumatological setting, generally have such a suggestive clinical and serological profile that performing a Surgical Lung Biopsy (SLB) becomes unjustifiable. Actually, SLB is associated with an in-hospital mortality of 1.7% and 16% for elective and non-elective procedures, respectively, and the underlying CTD is also considered a risk factor for mortality [75]. However, as already reported, in respiratory settings, a significant proportion of patients with autoimmune diseases can present with lung manifestations as the first, main, or even sole clinical feature of the disease, raising major diagnostic issues. In any case, obtaining histological samples for therapeutic purposes, also through cryobiopsy, which has demonstrated an acceptable diagnostic yield with a superior safety profile compared to SLB, could open up new options for diagnostic algorithms in real-world clinical practice [78].

Moreover, it is possible that the immunosuppressive treatment of UIP patients may produce different results, based on the specific underlying SARDs. For example, while for RA-UIP cases, immunosuppressive treatment could be useful, in SSc-UIP, current indications are limited. Several studies have evaluated a sub-analysis of UIP-CTDs, but the results could have been influenced by the prevalence of RA-UIP cases. Also in these cases, histological evaluation can be useful, at least for research purposes, in order to improve knowledge on this condition.

## 9. Conclusions

In conclusion, at present, there is no clear evidence that immunosuppressive treatments could be effective in UIP patients with autoimmune rheumatic disease. In all likelihood, considering that different results were potentially related to different stages of the disease, more frequent histological characterization could allow for a better understanding of these conditions and, ultimately, improve their therapeutic management. Using an aggressive diagnostic approach could reduce diagnostic uncertainties related to incomplete clinical presentations of autoimmune diseases in UIP patients, resulting in homogeneous enrollment in research studies. Specific clinical trials are required to assess the actual role of immunosuppressive treatment in these patients.



**Bullet-point conclusions**

There is a need for clinical trials specifically designed to assess the role of immunosuppression in secondary UIP.Despite an autoimmune pathogenesis, it is not possible to exclude the possi-bility that different conditions have different responses to immunosuppres-sion in the context of a UIP pattern.Histological lung samples in autoimmune ILD can be useful for diagnostic, therapeutic and research purposes.



## Figures and Tables

**Table 1:** Summarization of the cited studies.

Immunosuppression on UIP-SARDs					
Ref.	Condition	Patients	UIP	Treatment	Results
[60]	CTD	93	30	AZA, MMF	The two treatments were similar for FVC stabilization and adverse effects
[61]	CTD	125	32	MMF	Stabilization of FVC
[62]	CTD	49	20	RTX	Stabilization of FVC
[63]	CTD	26	11	RTX	Stabilization of FVC
[59]	SSc	41	15	MMF	Trend of decline compared with placebo
[36]	SSc	22	8	CYC	Trend of increased mortality for UIP compared with NSIP
[64]	RA	70	50	Non-antiTNF biological drugs	Significantly reduced risk of worsening for UIP
[65]	RA	31	13	RTX	Increased FVC
[66]	RA	56	20	RTX	Significant progression of lung damage; UIP a risk factor for mortality
[67]	RA	28	15	RTX	Significant decrease in lung function and higher mortality compared with other patterns
[68]	RA	78	78	TOFA	Significant reduction in progression
[69]	RA	42	18	JAKi	Stabilization of FVC
[70]	RA	212	80	RTX, AZA, MMF	Stabilization of FVC
[71]	IPAF	56	56	AZA, MMF	Greater proportion of improved/stable patients compared with anti-fibrotics

Legend: AZA: Azathioprine; CTD: Connective Tissue Disease; CYC: Cyclophosphamide; FVC: Forced Vital Capacity; IPAF: Interstitial Pneumonia with Autoimmune Features; JAKi: Jak inhibitors; MMF: Mycophenolate Mofetil; RA: Rheumatoid Arthritis; RTX: Rituximab; SSc: Systemic Sclerosis; and TOFA: tofacitinib.

## Data Availability

Not applicable.

## Abbreviations

The following abbreviations are used in this manuscript:

ACPA	Anti-Citrullinated Protein Antibody
AEC1	Alveolar Epithelial Type I Cells
AEC2	Alveolar Epithelial Type II Cells
ANCA	Anti-Neutrophil Cytoplasmic Antibodies
ASyS	Anti-Synthetase Syndrome
AZA	Azathioprine
CTD	Connective Tissue
CTGF	Connective Tissue Growth Factor
FVC	Forced Vital Capacity
GGO	Ground Glass Opacities
GPA	Granulomatosis with Polyangiitis
HC	Honeycombing
HRCT	High-Resolution Computed Tomography
IIM	Idiopathic Inflammatory Myopathies
ILD	Interstitial Lung Disease
IPAF	Interstitial Pneumonia with Autoimmune Features
IPF	Idiopathic Pulmonary Fibrosis
LIP	Lymphocytic Interstitial Pneumonia
MMF	Mycophenolate Mofetil
MPA	Micropolyangiitis
NSIP	Nonspecific Interstitial Pneumonia
OP	Organizing Pneumonia
pSS	Primary Sjögren’s Syndrome
RA	Rheumatoid Arthritis
RTX	Rituximab
SARDs	Systemic Autoimmune Rheumatic Diseases
SLB	Surgical Lung Biopsy
SSc	Systemic Sclerosis
TGFβ	Transforming Growth Factor β
UIP	Usual Interstitial Pneumonia
